# Numerical Study of the Influence of Coupling Interface Emissivity on Aerogel Metal Thermal Protection Performance

**DOI:** 10.3390/gels7040250

**Published:** 2021-12-03

**Authors:** Fengfei Lou, Sujun Dong, Yinwei Ma, Bin Qi, Keyong Zhu

**Affiliations:** 1 School of Aeronautical Science and Engineering, Beihang University, Beijing 100191, China; lloufengfei@163.com (F.L.); dsj@buaa.edu.cn (S.D.); 2 Research Department of Airframe Technology, Beijing Institute of Aerospace Technology, Beijing 100074, China; riskyma@163.com; 3 Qian-Xuesen Laboratory of Space Technology, China Academy of Space Technology, Beijing 100094, China; qionline@163.com

**Keywords:** metal thermal protection system, aerogel, thermal conductivity, emissivity, radiative heat transfer

## Abstract

For aerogels in metal thermal protection system (MTPS), radiative heat transfer will participate in the thermal transport process. Therefore, the influence of the emissivity of the coupling interface between metal and aerogels on thermal insulation performance is considered an important research focus. In this paper, CFD numerical simulation is performed to study the influence of emissivity on the performance with different extinction coefficients at different boundary temperatures. The finite volume method and the discrete ordinate method are used to solve the govern equations. The results show that when the boundary temperatures are 600 K and 2100 K, the extinction coefficient is 50 m^−1^, and the reduction percentage of the effective thermal conductivity with an emissivity of 0.2 can be up to 47.5% and 69.8%, compared to the system with an emissivity of 1. Thus, the reduction in emissivity has a good effect on the thermal insulation performance of the MTPS at a higher boundary temperature for materials with small extinction coefficients.

## 1. Introduction

Thermal protection systems are there to ensure the safety of hypersonic flight vehicle work in extreme conditions [1]. When the vehicle is flying at a hypersonic speed, its surface has severe friction with the atmosphere. Severe aerodynamic heating will make the surface temperature rise sharply, and the local temperature may even exceed 1800 K, resulting in the rise in the internal temperature of the vehicle, which will lead to the degradation of material and structural properties [2].

A metal thermal protection system (MTPS) can be used in space shuttles exposed to high-temperature environments for a long time because it is reusable, lightweight and has good impact resistance. Thermal insulation materials play a very important part in this. Among them, lightweight and efficient super insulation materials, such as aerogels and their composites, have attracted more and more attention and application in recent years [3]. The thermal conductivity of thermal insulation materials is a parameter reflecting their thermal performance, and it is the main identifier of materials’ excellent thermal insulation performance. However, for porous, semi-transparent media such as aerogels and their composites, radiative heat will be absorbed, scattered and re-emitted in a solid medium [4]. For the existing technology of measuring thermal conductivity, there is a deviation between the test theory and the actual heat transfer process, so the measurement of the thermal conductivity of porous, semi-transparent materials is still an urgent research topic.

There are two kinds of thermal conductivity test methods: steady-state methods and transient-state methods [5]. Steady-state methods contain the heat flow meter method, the hot plate method, etc. These methods are based on the one-dimensional (1D) Fourier law of heat conduction [6]. Although steady state methods have the advantages of direct testing and high accuracy, large test sample sizes are required and are time consuming. On the contrary, transient methods, such as the hot wire (HW) method, hot strip (HS) method, laser flash method and transient plane source (TPS) method, only require small test sample sizes and short test times [7]. Coquard et al. [8] investigated the possibility of applying the laser flash method, HW method [9] and TPS method [10] to semi-transparent materials. However, their conclusion does not have generality since the analysis was only conducted at room temperature where radiation is not the dominant heat transfer process and can usually be neglected. Zhang et al. [11,12] studied the effect of radiative heat transfer on determining the thermal conductivity of semi-transparent materials using HW and TPS. Because there is no specific formula to calculate the thermal conductivity of the system, the thermal conductivity calculated by the one-dimensional (1D) Fourier steady-state method is used as the real thermal conductivity.

Based on the study on the performance of MTPS, the influence of the emissivity of the coupling interface between metal and aerogels on the performance of MTPS is considered another research focus. Because the emissivity of the coupling interface will directly affect the radiative heat transfer in the aerogel medium, the effects of different coupling interface emissivity values on the thermal conductivity of MTPS is worthy of being studied. The relationship between emissivity and radiative heat transfer has been studied in many fields by scholars at home and abroad. Jabbari et al. [13] calculated the heat transfer rate and emissivity of surfaces when one and two thick radiation shields are placed between two thick spheres. The calculations showed that the use of a radiation shield with a lower emissivity was better than two radiation shields with a higher emissivity at reducing the heat transfer rate. Zu et al. [14] proposed that the high-emissivity coating enhanced the heat transfer of passive heat sinks and had a promising prospect as an excellent thermal management material. Saravanan and Raja [15] discussed the problem of thermal radiation in the presence of nonuniform emissivity arising through different types of surfaces involved in thermal-control systems. The results showed that prominent heat transfer enhancement occurs when the emissivity of the inner hot plate is higher.

The relationship between emissivity and radiative heat transfer has been studied in the above articles, and the following typical studies have been conducted in MTPS. Tan et al. [16] proposed that thermal energy can be reduced via increasing the radiation and conduction heat transfer away from the surface, so an emissivity modifier was incorporated into an ultra-high-temperature ceramic coating system (ZrB_2_/SiC) to increase its surface radiation heat transfer rate by increasing the emissivity of the surface in the thermal protection system. Chen et al. [17] studied a compound multi-layer insulation structure which was proposed for use in high-temperature cylinder thermal protection systems. The thermal insulation performance was much better when the emissivity of the system was higher. Ji et al. [18] designed a multi-layer thermal insulation in thermal protection systems and found that when the density of insulation materials and the emissivity of the outer surface of foils were higher, the temperature of the bottom surface was lower.

From the above research, it can be seen that the research on emissivity by scholars mainly focuses on the relationship between surface emissivity and radiative heat transfer. Even for MTPS, the emphasis is still on the effect of the emissivity of the outer surface on the thermal insulation performance of the system. However, in the MTPS, radiation heat will participate in the heat transfer due to the unique heat transfer mechanism of aerogel, so it is very important to study the coupling interface emissivity between metal and aerogel for the thermal insulation performance of the MTPS. However, when Zhang [11,12] studied the effect of radiative heat transfer on determining the thermal conductivity of semi-transparent materials using HW and TPS, the results showed that the extinction coefficient and the boundary temperature of the system had great influence on the accuracy. Therefore, the influence of the extinction coefficient and boundary temperature must be considered when studying the thermal insulation performance of MTPS with different coupling interface emissivity values.

In this paper, the effect of coupling interface emissivity on the performance of MTPS is numerically analyzed, which has never been studied in this field. In this research process, due to the involvement of radiative heat transfer in an aerogel medium, no specific formula has been used to calculate the thermal conductivity of MTPS. Thus, the 1D Fourier steady-state thermal conductivity calculation method is used to calculate the thermal conductivity [11,12]. According to the Stefan–Boltzmann law [19], the radiation emission of the coupling interface is proportional to T^4^, so the boundary temperature of the MTPS will indirectly affect the radiation emission of the coupling interface. For a semi-transparent medium, radiation will participate in the heat transfer process, which is affected by the extinction coefficient (absorption coefficient and scattering coefficient) of the materials. Based on the above reasons, the effective thermal conductivity (ETC) is mainly affected by the extinction coefficient of a semi-transparent medium, the boundary temperature of MTPS and the emissivity of the coupling interface. Therefore, the purpose of this paper is to study the influence of coupling interface emissivity on the performance of MTPS with different extinction coefficients at different boundary temperatures.

## 2. Results and Discussion

The research focus of this paper is to study the effect of variations in the coupling interface emissivity on the performance of MTPS, which is very significant. Because the emissivity of a highly polished metal surface is very small for the same metal material, the effect of the degree of surface polishing (i.e., the degree of the reduction in the coupling interface emissivity) on the thermal insulation performance of MTPS should be given sufficient attention. In order to evaluate the influence of the variations in coupling interface emissivity on the performance of MTPS, the emissivity varies from 0.2 to 1.

### 2.1. The Influence of Emissivity Variation with Different Extinction Coefficients

**Figure 1:**
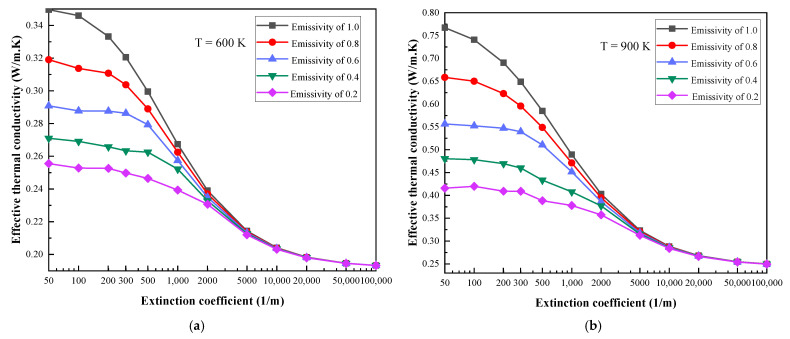

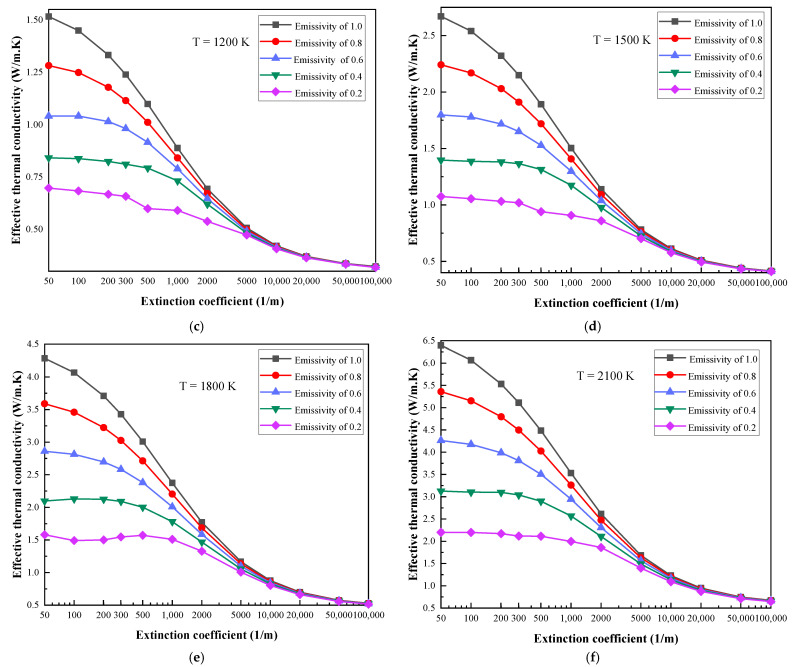
The variation in ETC values with different extinction coefficients: (**a**) T = 600 K, (**b**) T = 900 K, (**c**) T = 1200 K, (**d**) T = 1500 K, (**e**) T = 1800 K, (**f**) T = 2100 K.

Figure 1 shows the change of the ETC of the MTPS with the extinction coefficients of the aerogel at the boundary temperatures of 600 K, 900 K, 1200 K, 1500 K, 1800 K and 2100 K. In each diagram, the ETC is calculated and compared at five different emissivity values of the coupling interface, which are 0.2, 0.4, 0.6, 0.8 and 1, respectively. The trend in the variations in the ETC with the extinction coefficient is basically the same at six boundary temperatures and five different coupling interface emissivity values.

As shown in Figure 1a, when the boundary temperature is 600 K, the emissivity is 1, the extinction coefficients are 50 m^−1^ and 100,000 m^−1^, and the ETC values are 0.3499 W/m. K and 0.1933 W/m. K, respectively. It can be seen that when the extinction coefficient increases from 50 m^−1^ to 100,000 m^−1^, the ETC can be reduced by 44.76%. Under the same premise, when the boundary temperature is 2100 K, the reduction percentage is 89.47%. In addition, there is still the same trend under different boundary temperatures and emissivity; that is, the ETC decreases with increases in the extinction coefficient.

Regarding this research result, Zhang [11,12] also reached a similar conclusion when studying the influence of radiation on the thermal conductivity of semi-transparent materials measured by TPS and HW. The measurement error decreases with the increase in the extinction coefficient, which is mainly because the material with a larger extinction coefficient has an enhanced suppression of thermal radiation. Based on Figure 1, a more profound explanation will be given. The main reason for the result is that when the extinction coefficient is larger than 5000 m^−^^1^ (optical thickness ≥ 50; optical thickness t=β×δ; β is the extinction coefficient of the aerogel; and δ is the thickness of aerogel), the semi-transparent medium has been recognized as an optical thick medium [20]. The optical thick medium assumes that thermal radiation only penetrates a very short distance [21,22]. Thus, in the optical thick material, the radiative heat transfer can be regarded as a thermal diffusion process and the effect of radiative heat transfer is small. This is the reason why the ETC changes slowly with the increase in the extinction coefficient when the extinction coefficient is greater than 5000 m^−^^1^. When the extinction coefficient is less than this value, the system is greatly affected by radiation, so changes in ETC are very sensitive to changes in the extinction coefficient.

**Figure 2:**
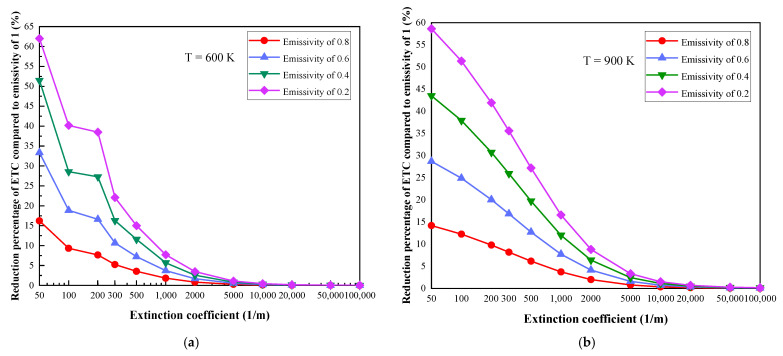

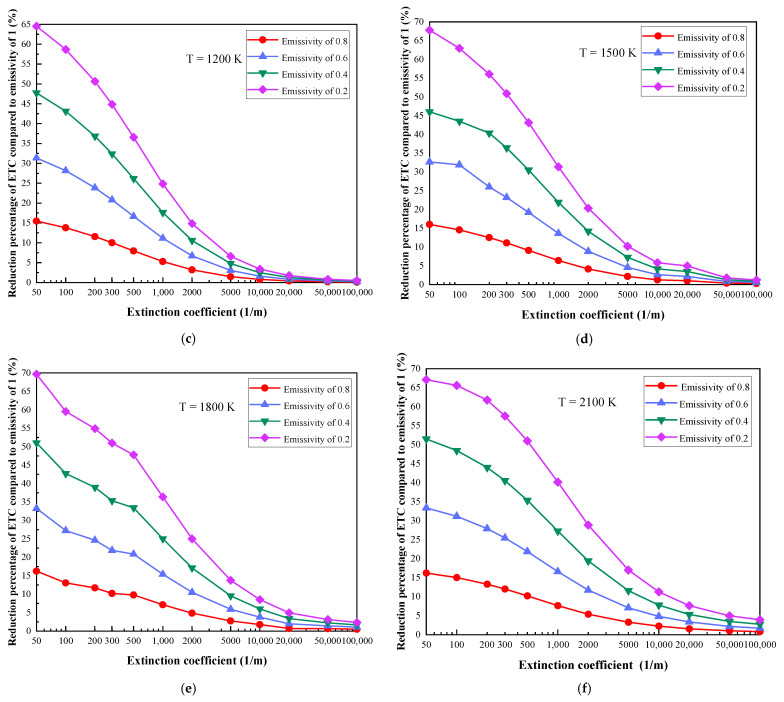
Reduction percentage of ETC compared to the system with an emissivity of 1: (**a**) T = 600 K, (**b**) T = 900 K, (**c**) T = 1200 K, (**d**) T = 1500 K, (**e**) T = 1800 K, (**f**) T = 2100 K.

The research focus is the influence of coupling interface emissivity variations on the performance of MTPS. As shown in Figure 2, at specific boundary temperatures, the reduction percentage of the ETC relative to the system with an emissivity of 1 decreases with the increase in the extinction coefficient. At the same time, under the conditions of specific boundary temperatures and extinction coefficients, the reduction percentage of ETC increases with the decrease in emissivity. It can be seen from Figure 2f that when the boundary temperature is 2100 K, the extinction coefficients are 50 m^−1^ and 100,000 m^−1^, the ETC with emissivity values of 0.8, 0.6, 0.4 and 0.2 are reduced by 16.22%, 33.36%, 51.49%, 69.8% and 0.82%,1.72%, 2.76%, 3.94%, respectively, compared to the system with an emissivity of 1.

It can be seen from Figure 2 that the change in the trend of the coupling interface emissivity values on the thermal insulation performance is basically the same as that in Figure 1. When the extinction coefficient is greater than 5000 m^−1^, changes in emissivity have little influence and can even be ignored; however, when it is less than 5000 m^−1^, it changes greatly, and the reason can still be explained by using an optical thick medium. The conduction and radiation coupled heat transfer process within the thermal insulation material can be regarded as a diffusion process when the extinction coefficient is large, and the influence of coupling interface emissivity can be ignored in this circumstance. However, for materials with small extinction coefficients, the thermal radiation penetrates a longer distance, so the decrease in the emissivity of the coupling interface has a great effect on the thermal insulation performance, and the reduction percentage of ETC can be up to 69.8%. Under this condition, the thermal insulation performance of the MTPS can be greatly improved by highly polished metal.

**Figure 3:**
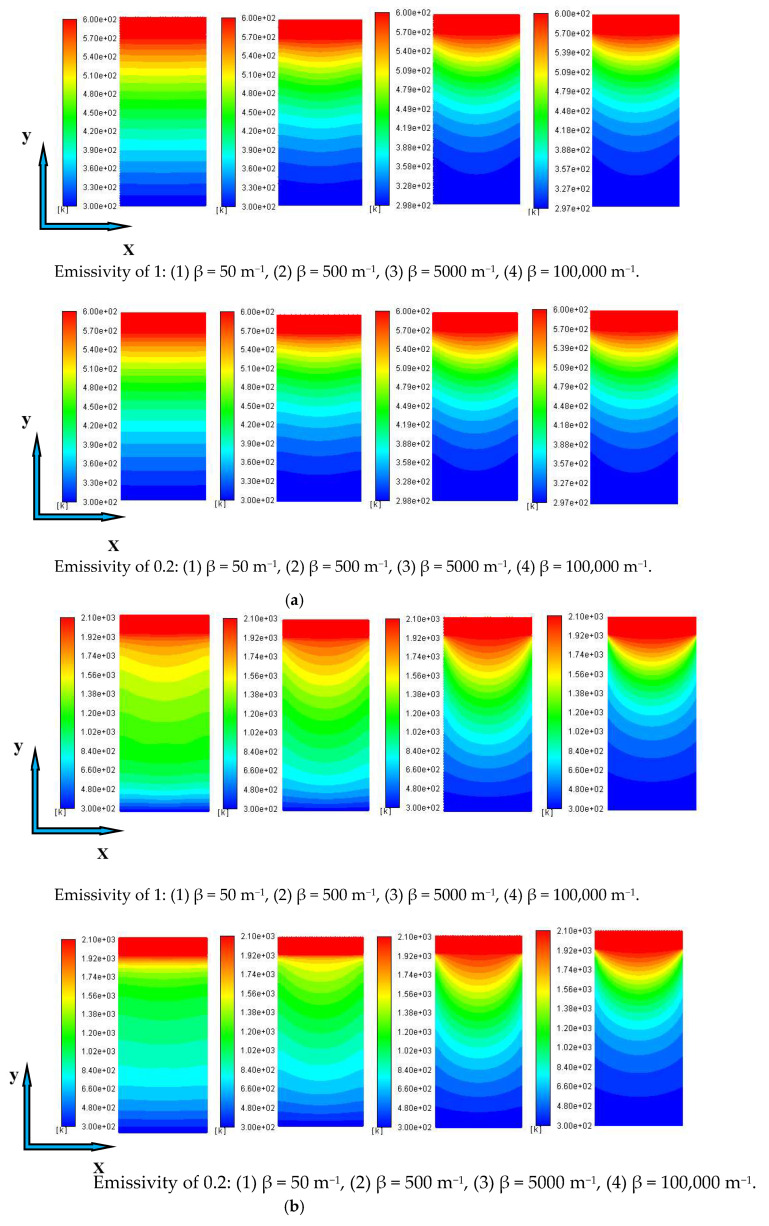
Temperature diagrams with extinction coefficients at emissivity values of 1 and 0.2: (**a**) T = 600 K, (**b**) T = 2100 K.

**Figure 4:**
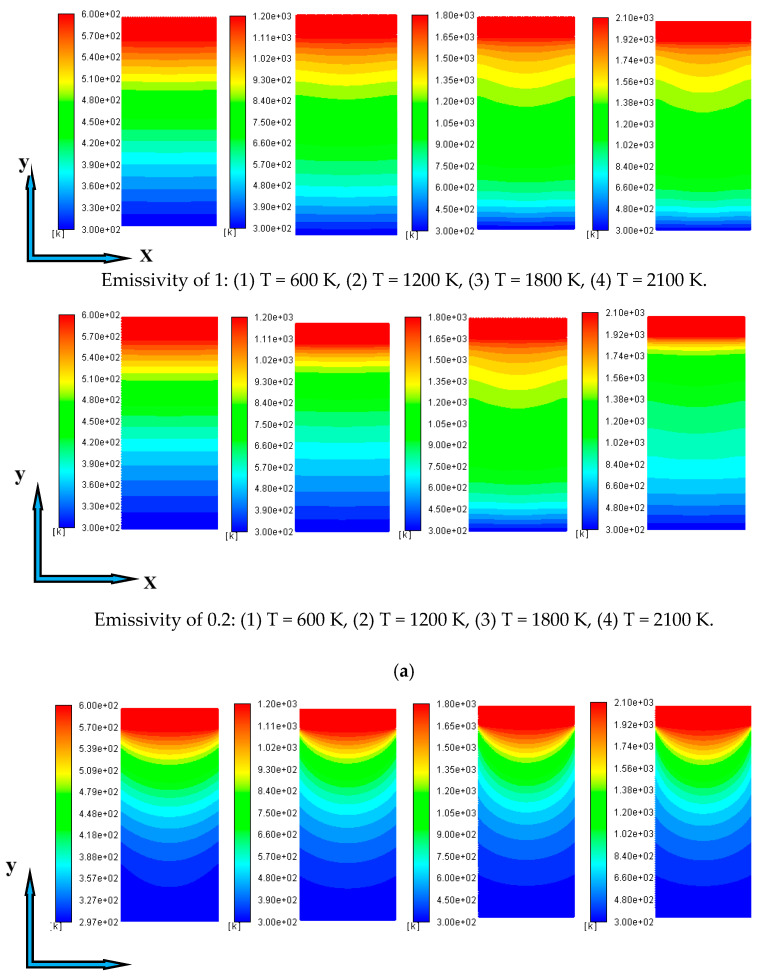

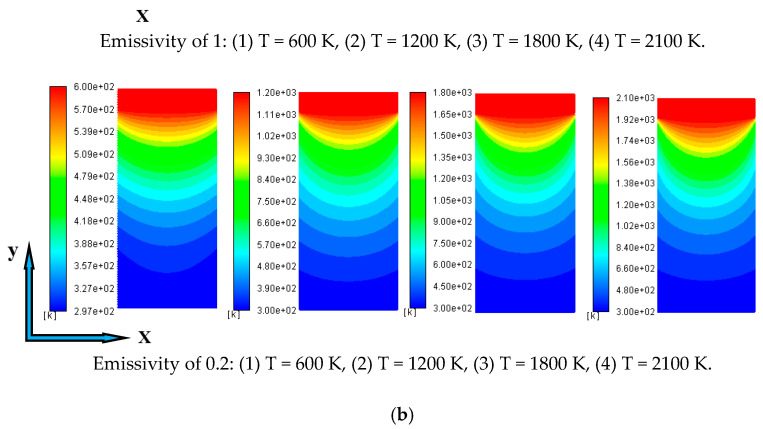
Temperature diagrams with different boundary temperatures at the emissivity of 1 and 0.2: (**a**) β = 50 m^−1^, (**b**) β = 100,000 m^−1^.

Under the premise that the boundary temperatures are 600 K and 2100 K, and the emissivity values of the coupling interface are 1 and 0.2, the temperature diagrams with changes in the extinction coefficient are shown in Figure 3. With the increase in the extinction coefficient, the dispersion behavior of temperature becomes more obvious. Figure 4 shows the change in temperature diagrams with the boundary temperatures at extinction coefficients of 50 m^−1^ and 100,000 m^−1^, respectively.

When the extinction coefficient is 50 m^−1^, the temperature change is almost linear; however, when the extinction coefficient is greater than 5000 m^−1^ (optical thickness ≥ 50), the dispersion behavior of the temperature is basically the same. The reason can still be explained by the optical thick medium. When the extinction coefficient is large, the conduction and radiation coupling heat transfer process inside the material is considered a heat diffusion process. Although, for materials with small extinction coefficients, especially in high-temperature environments, the radiation penetration distance is long and the radiation heat transfer is dominant; thus, the manifestation of the temperature diagram is different from that of materials with large extinction coefficients.

### 2.2. The Influence of Emissivity Variation with Different Boundary Temperatures

**Figure 5:**
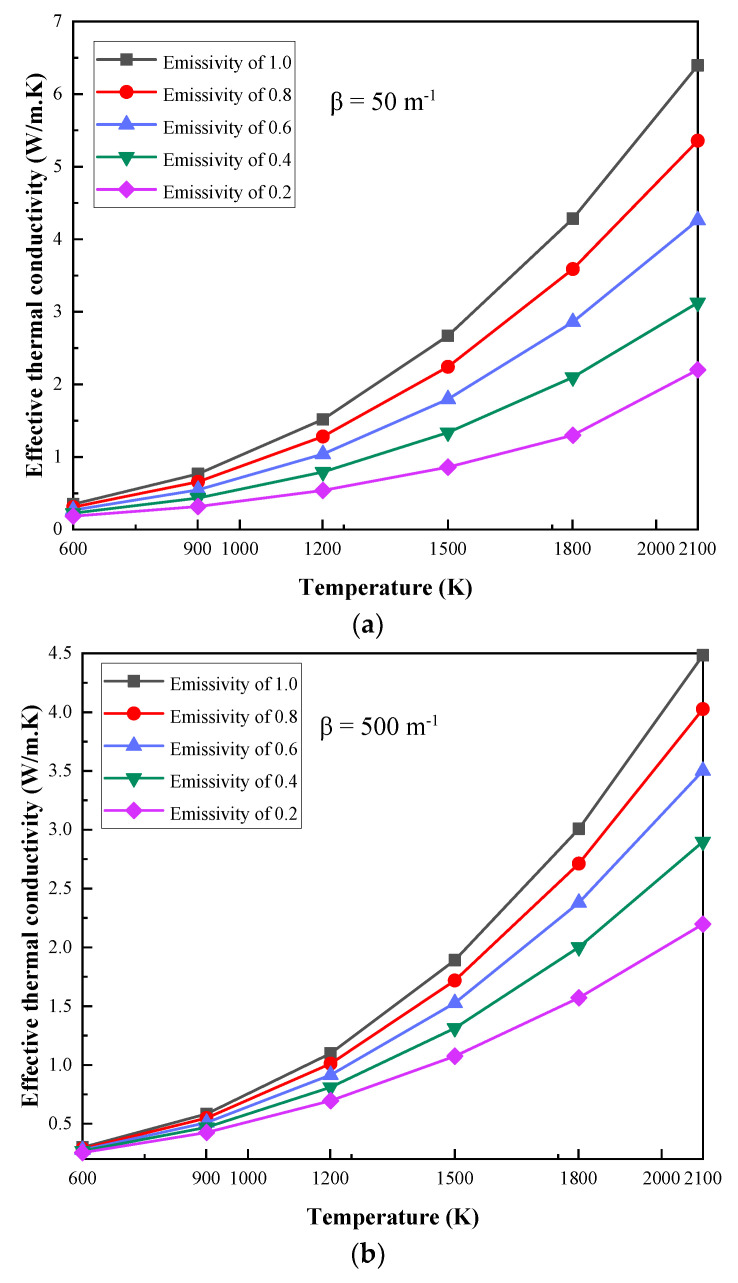

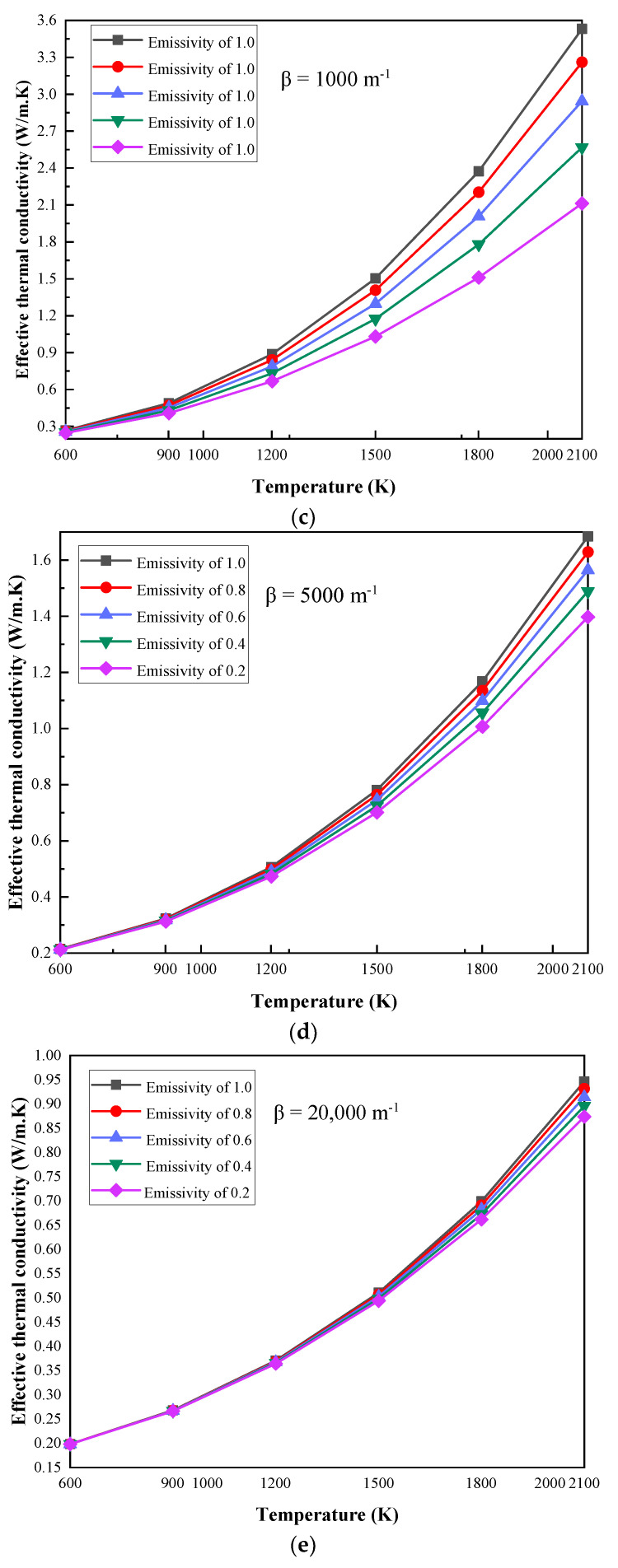

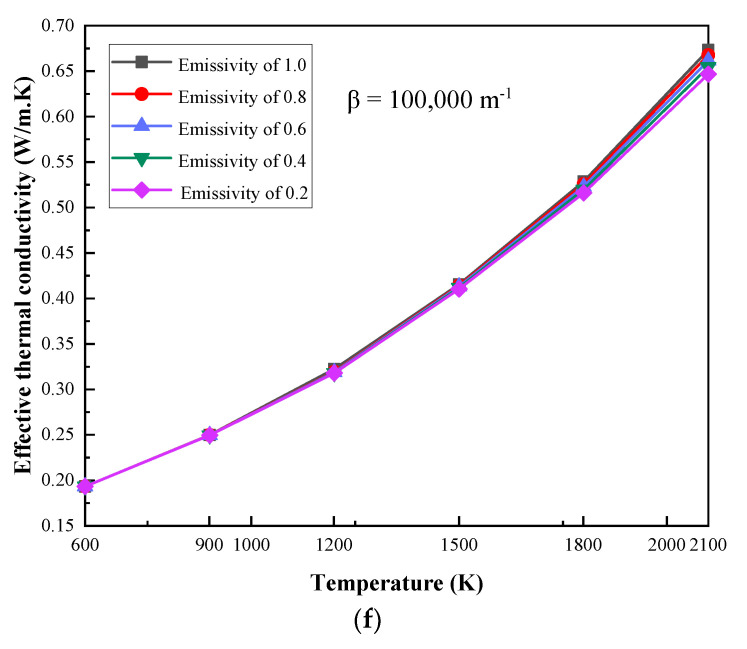
The variation in ETC with different boundary temperatures: (**a**) β = 50 m^−1^, (**b**) β = 500 m^−1^, (**c**) β = 1000 m^−1^, (**d**) β = 5000 m^−1^, (**e**) β = 20,000 m^−1^, (**f**) β = 100,000 m^−1^.

Figure 5 shows the change in the ETC of the MTPS with different boundary temperatures at extinction coefficients of 50 m^−1^, 500 m^−1^, 1000 m^−1^, 5000 m^−1^, 20,000 m^−1^ and 100,000 m^−1^. In each diagram, the ETC is calculated and compared at five different emissivity values of the coupling interface, which are 0.2, 0.4, 0.6, 0.8 and 1.

According to Figure 5a, when the extinction coefficient is 50 m^−1^, the emissivity is 1, and the boundary temperatures are 600 K and 2100 K, the ETC values are 0.3496 W/m. K and 6.3958 W/m. K, respectively, which shows that the ETC increases greatly. Under the same premise, when the extinction coefficient is 100,000 m^−1^, the ETC values are 0.1933 W/m. K and 0.6732 W/m. K, respectively, with a small increase. It can be seen from Figure 5 that when the boundary temperature is larger than 1500 K, the rate of increase of the ETC is faster, and the change in trends is more obvious in the case of low extinction coefficients. In addition, there is still the same trend under different extinction coefficients and emissivity values; thus, the ETC increases with the increase in the boundary temperature.

**Figure 6:**
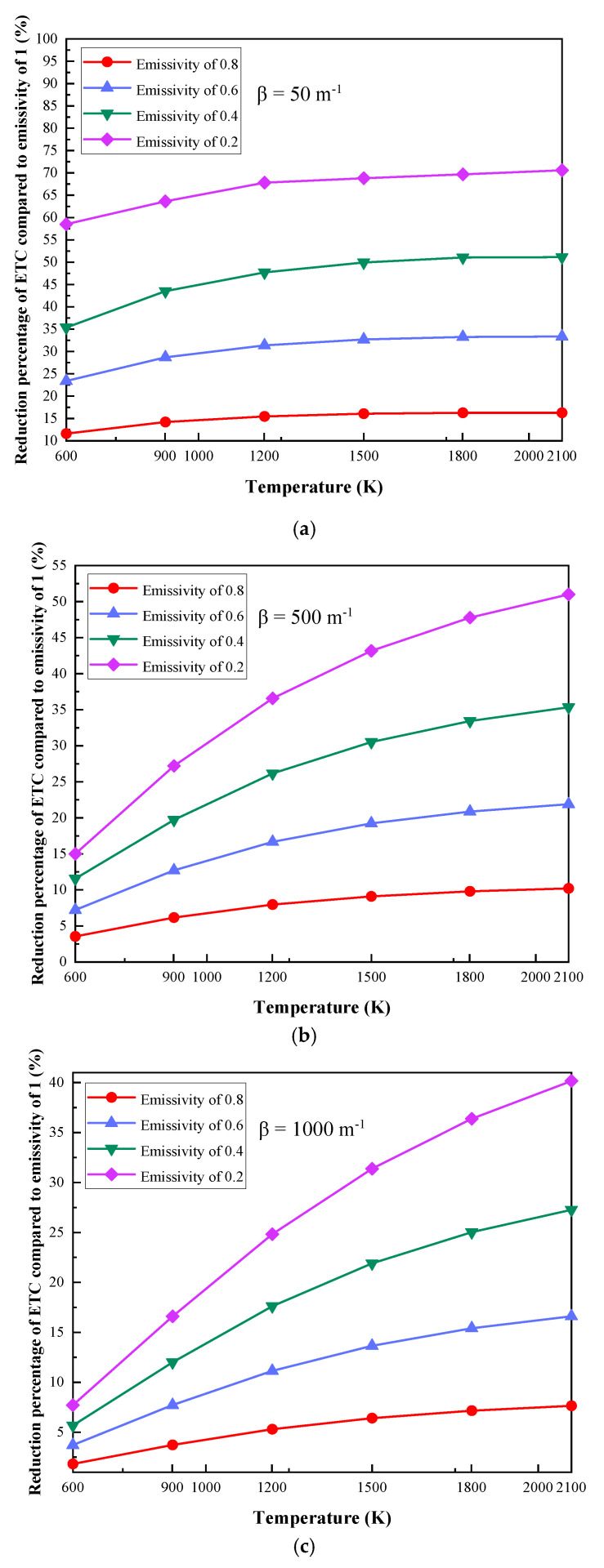

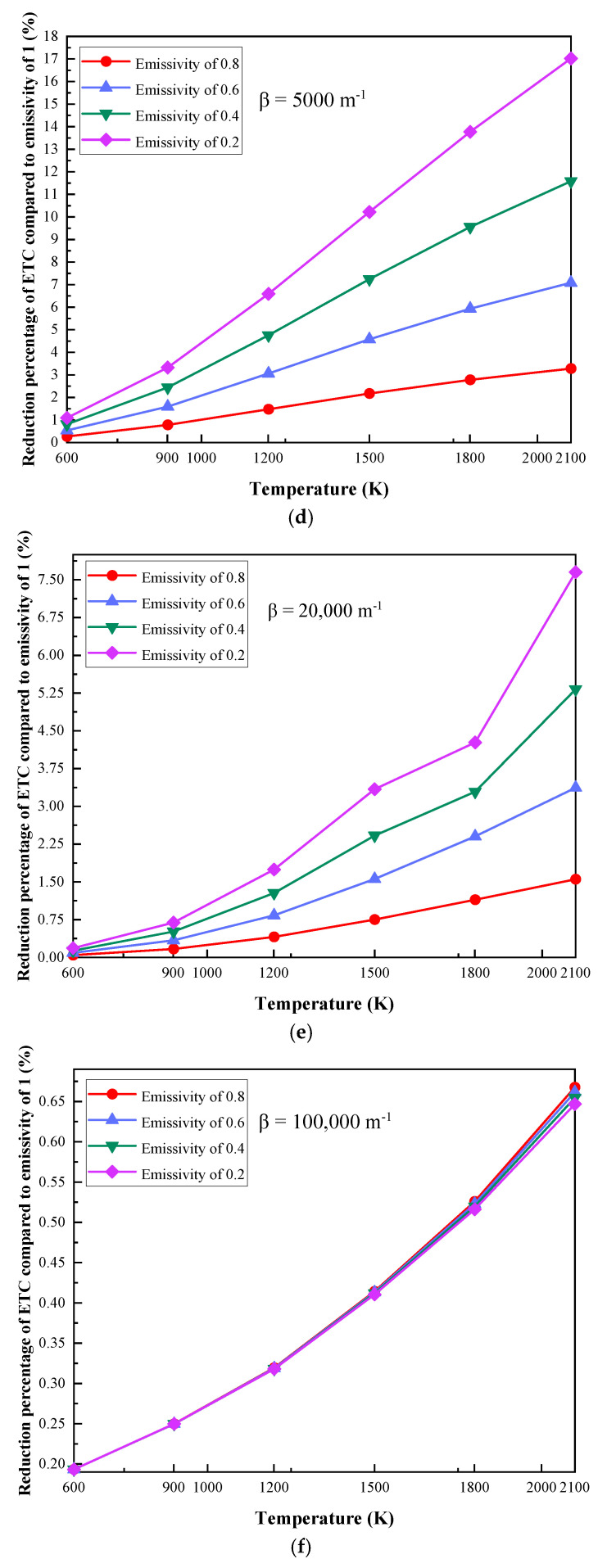
Reduction percentage of ETC compared to an emissivity of 1: (**a**) β = 50 m^−1^, (**b**) β = 500 m^−1^, (**c**) β = 1000 m^−1^, (**d**) β = 5000 m^−1^, (**e**) β = 20,000 m^−1^, (**f**) β = 100,000 m^−1^.

As shown in Figure 6, at specific extinction coefficients, the reduction percentage of the ETC at different emissivity values increases with the increase in the boundary temperature compared to the system with an emissivity of 1. When the extinction coefficient is 50 m^−1^, the boundary temperatures are 600 K and 1500 K, and the ETC with emissivity values of 0.8, 0.6, 0.4 and 0.2 are reduced by 11.61%, 23.39%, 35.36%, and 47.5% and 16.05%, 33.69%, 40.36%, and 67.8%, respectively.

In short, at specific extinction coefficients, the ETC increases with the increase in the boundary temperature. At the same time, compared to the system with an emissivity of 1, the reduction percentage of ETC has the same trend. Moreover, when the boundary temperature is larger than 1500 K, the effect of the emissivity variation of the coupling interface on the performance is significantly improved.

According to the Stefan–Boltzmann law [19], the radiation emission is proportional to T^4^. Because the boundary temperature of the MTPS will indirectly affect the temperature of the coupling interface, the higher boundary temperature has a greater influence on the radiative heat transfer, and the reduction in emissivity has a good effect on the thermal insulation performance of the MTPS in this circumstance. This is also the reason why when the boundary temperature is larger than 1500 K, the effect of the emissivity variation of the coupling interface on the performance is significantly improved. In MTPS, this research focus has never been studied, and most articles focus on the influence of outer surface emissivity of MTPS on thermal insulation performance [16,17,18]. The results show that when the emissivity of the outer surface is larger, the MTPS will have a larger radiation emission to the outside of the system and a lower boundary temperature; thus, the system will have a better heat insulation performance, which can prove the accuracy of the research content of this paper.

## 3. Conclusions

In this paper, the effect of coupling interface emissivity on the performance of MTPS is studied. Since the boundary temperature and extinction coefficient are also considered to be important factors, the influence of emissivity on the performance with different extinction coefficients at different boundary temperatures is numerically analyzed. The main conclusions are as follows:

(1) At specific boundary temperatures, the ETC decreases with the increase in the extinction coefficient. When the extinction coefficient increases from 50 m^−1^ to 100,000 m^−1^, ETC with an emissivity of 1 can be reduced by 44.76% and 89.47% at boundary temperatures of 600 K and 2100 K, respectively.
(2) Compared to the system with an emissivity of 1, the reduction percentage of the ETC decreases with the increase in the extinction coefficient and increases with the decrease in emissivity at specific boundary temperatures. When the boundary temperature is 2100 K, the extinction coefficients are 50 m^−1^ and 100,000 m^−1^, and the ETC with emissivity values of 0.8 and 0.2 are reduced by 16.22% and 69.8%, and 0.82% and 3.94%, respectively.
(3) For materials with small extinction coefficients, the decrease in emissivity has a great effect on the thermal insulation performance, and the reduction percentage of ETC can be up to 69.8%. However, when the extinction coefficient is large, the influence of emissivity variation can be ignored in this situation.
(4) The ETC increases with the increase in the boundary temperature at specific extinction coefficients. When the boundary temperatures are 600 K and 2100 K, the ETC values are 0.3496 W/m. K and 6.3958 W/m. K at the extinction coefficient of 50 m^−1^ and emissivity of 1, respectively. This shows that the ETC increases greatly.
(5) The reduction percentage of the ETC increases with the increase in the boundary temperature. When the extinction coefficient is 50 m^−1^, the boundary temperatures are 600 K and 1500 K, and the ETC with emissivity values of 0.8 and 0.2 are reduced by 11.61% and 47.5%, and 16.05% and 67.8%, respectively.
(6) Higher boundary temperatures have a greater influence on the radiative heat transfer, and the reduction in emissivity has a good effect on the thermal insulation performance of the MTPS in this situation.

## 4. Methodology and Preparation Work

### 4.1. Methodology

In order to evaluate the influence of the coupling interface emissivity on the performance of MTPS, the “numerical experiment” method is used to simulate the experimental process. The research steps of the paper are mainly divided into two steps to complete the research objective.

The first step was to verify the correctness of the simulation. Firstly, because the commercial software ICEM was used to generate a grid, grid independence verification needs to be given. Secondly, in the simulation process, the density ρ, the thermal conductivity of conduction λ, the extinction coefficient β, the specific heat capacity c, refractive index n and inner emissivity ε’ of aerogel are given. Taking the coupling interface of MTPS as the research object, the radiation emission from the coupling interface obtained by the “numerical experiment” is compared with the calculated value, and the calculation formula is shown in formula (1). Theoretically, the simulated value is equal to the calculated value, so the correctness of the simulation can be verified again.

(1) $$q_{R}=n^{2}\varepsilon \sigma T_{p}^{4}$$

where $q_{R}$ is the radiation emission from the coupling interface, n is the refractive index of the aerogel, ε is the emissivity of the coupling interface, σ is the Stefan–Boltzmann constant, and $T_{p}^{4}$ is the temperature of the coupling interface.

The second step is to study the influence of coupling interface emissivity on the performance of MTPS with different extinction coefficients of semi-transparent media at different boundary temperatures. For semi-transparent media, radiation will participate in heat transfer, which is affected by the extinction coefficient (absorption coefficient and scattering coefficient) of aerogel, so there is no specific formula to calculate the real thermal conductivity of MTPS.

Based on the above problems, the 1D Fourier steady-state thermal conductivity calculation method is used to calculate the thermal conductivity of MTPS [11,12], which is referred to as the ETC. The 1D steady coupling heat transfer equation via conduction and radiation and the radiative transport equation are also solved using the finite volume method (FVM) and the discrete ordinate method (DOM), respectively. With given parameters such as the extinction coefficient, the fixed temperature of hot face (boundary temperature) and the fixed temperature of cold face (300 K), the ETC can be calculated from the following formula by simulating the total heat flux.

(2) $$\lambda =q_{t}\delta ’/\Delta T$$

where $q_{t}$ and ΔT are the total heat flux and temperature difference across the MTPS and δ’ is the overall thickness of the MTPS. The ETC obtained from the 1D steady state calculation method is regarded as the true thermal conductivity of MTPS.

### 4.2. Preparation Work

In this section, the influence of the emissivity variations of the coupling interface on the performance of grey, pure absorptive semi-transparent aerogel without scattering is investigated.

#### 4.2.1. Verification of the Simulation

Firstly, the mesh independence check of the two-dimensional (2D) model was carried out in order to determine the appropriate mesh size. The ETC obtained from the 1D steady-state method under different grid sizes was calculated on the premise that the specific values of the thermal conductivity of conduction, density, specific heat capacity, refractive index, extinction coefficient and the inner emissivity of metal and aerogel were given. The results show that when the grid size is larger than 143,687, the ETC obtained by the 1D steady-state method basically does not differ; thus, the grid system with the size of 286,346 was selected in the following simulation.

Secondly, the grid system with a size of 286,346 was selected in the following verification simulation. Under certain working conditions, the radiation emission from the coupling interface is obtained by simulation, which is compared with the calculated value. The result shows that the simulated value is consistent with the calculated value, which proves the correctness of the simulation again.

#### 4.2.2. Computational Domain and Boundary Conditions

**Figure 7:**
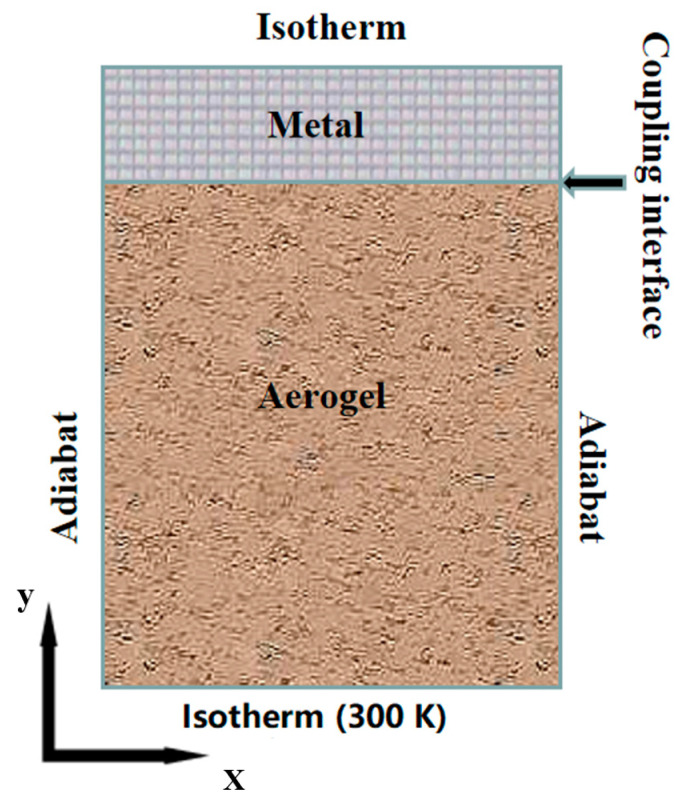
Schematic diagram of computation domain.

As shown in Figure 7, the computational domain is simplified to a 2D heat transfer problem. The size is 5 mm in length and 11 mm in thickness, of which the thickness of the metal area is 1 mm, and the thickness of the aerogel area is 10 mm. The boundary conditions are adiabatic on the side of the system and isothermal on the cold and boundary surfaces. In the simulation, the cold surface temperature $T_{C}$ was set to 300 K, the boundary temperatures $T_{H}$ were set to six different values, the extinction coefficients were set to twelve different values, and the emissivity values of the coupling interface were set to five different values. To make the calculation conditions clearer, the specific parameters are shown in Table 1. In addition, the physical properties of the materials are shown in Table 2.

In terms of physical parameters, the refractive indexes of metal and aerogel were set as 1.6 and 1.05, respectively [23,24]. Since the inside of metal does not participate in radiative heat transfer, the internal emissivity can be ignored. Meanwhile, the internal emissivity ε’ of the side and bottom aerogel was set as 0.5 [11]. In the calculation conditions, the emissivity of the coupling interface had five values, which were 0.2, 0.4, 0.6, 0.8 and 1. Emissivity is a parameter which indicates the ability of heat radiation emission and absorption, and the larger the value is, the stronger the ability for heat radiation emission becomes. For the same metal material, the emissivity of highly polished surfaces is very small, whereas that of rough and oxidized surfaces is often several times that of polished surfaces [25,26]; therefore, it is very meaningful to study the influence of the polishing degree of metal surface (that is, the decreased degree of coupling interface emissivity) on the thermal insulation performance of MTPS. It must be pointed out here that the emissivity of the actual object is less than 1. The reason for setting the coupling interface emissivity of this model to 1 is mainly to conduct a quantitative analysis for the reduction percentage of ETC. Compared with the system with a coupling interface emissivity of 1, the influence of the emissivity reduction on the thermal insulation performance of the MTPS is analyzed.

## 5. Numerical Method

### 5.1. Govern Equations

Taking the aerogel MTPS as an example, the energy equation within semi-transparent materials is [10]:

(3) $$\rho c\frac{\partial T(x,y)}{\partial t}=-\nabla \cdot q_{t}=-\nabla \cdot q_{c}-\nabla \cdot q_{r}=\frac{\partial }{\partial x}\left(\lambda \frac{\partial T}{\partial x}\right)+\frac{\partial }{\partial y}\left(\lambda \frac{\partial T}{\partial y}\right)-\nabla \cdot q_{r}$$

where $q_{t}$, $q_{c}$, $q_{r}$ are the total heat flux, conductive heat flux and radiative heat flux, respectively. The radiative heat flux is related to the radiative intensity within the materials:

(4) $$q_{r}=q_{r,x}e_{x}+q_{r,y}e_{y}$$

(5) $$q_{r,x}=\int_{\Omega =4\pi }I\xi d\Omega$$

(6) $$q_{r,y}=\int_{\Omega =4\pi }I\eta d\Omega$$

(7) ξ=sinθcosφ

(8) η=sinθsinφ

where $q_{r,x}$ and $q_{r,y}$ are the components of radiative heat flux in x and y coordinates, respectively; ξ is the direction cosine along the x coordinate and η is the direction cosine along the y coordinate; θ and φ are the zenith angle and circumference angle, respectively; Ω is the solid angle; and I is radiative intensity.

Among them, $q_{t}$, $q_{c}$ and $q_{r}$ in Equations (3) and (4) are all vectors, which can be divided into components on the x coordinate and y coordinate axes according to the heat transfer model in this paper. Equations (5) and (6) represent the components of radiative heat flux in the x and y coordinates, respectively, where the radiative intensity needs to be solved.

The radiative intensity within aerogel is governed by the radiative transport equation (RTE) [9,27]; the RTE is shown in Equation (9).

(9) $$\frac{dI(r,s)}{ds}=-\beta I(r,s)+\kappa I_{b}(r)+\frac{\sigma_{s}}{4\pi }\int_{\Omega_{i}=4\pi }I(r,s_{i})\Phi \left(s_{i},s\right)d\Omega_{i}$$

where β, κ, $\sigma_{s}$ are extinction, absorption and scattering coefficients, respectively; $I_{b}(r)$ is the radiative intensity emitted by a black body; I(r,s) represents the radiative intensity of space position r and transmission direction s, which is a vector; $\Phi \left(s_{i},s\right)$ is the scattering phase function, which is the ratio of the scattering intensity in the **s** direction caused by incident radiation in the $s_{i}$ direction to the average scattering intensity in the 4π scattering space. Here, because RTE is related to space and direction, I(r,s), $I(r,s_{i})$ and $\Phi \left(s_{i},s\right)$ in Equation (9) are all related to direction, which are vectors.

**Figure 8:**
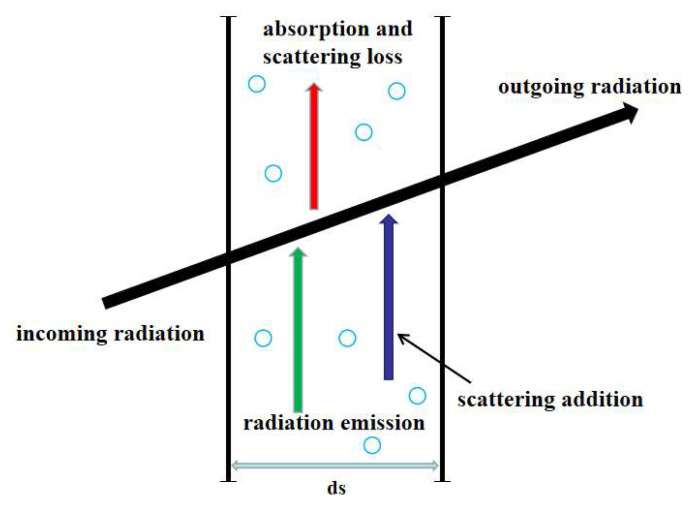
The schematic diagram of radiative heat transfer.

Figure 8 illustrates the process of radiative heat transfer. Based on RTE, the left side of the equation is the variation in the radiation intensity of a microelement in s direction at r position. The first term on the right is the attenuation of radiation intensity caused by absorption and scattering in the s direction; the second term is the enhancement of radiation intensity in the s direction caused by the radiation of the medium itself at r position; the third term is the sum of the scattering intensity enhancement in the s direction caused by the r point in the incident medium in all directions of 4π space (where the incident intensity in the $s_{i}$ direction is $I(r,s_{i})$).

### 5.2. Numerical Methods

It is necessary to know the radiative intensity to solve the heat flux field within the medium in Equation (3), which relies on solving Equation (9). Meanwhile, the heat flux field should be known to determine the radiative intensity field in Equation (9). Therefore, Equations (3) and (9) should be solved alternately until consistency is reached between the heat flux field and radiative intensity field at each time step. The energy equation is solved by FVM and the RTE is solved by DOM.

The FVM is based on the differential heat conduction equation, which can be directly obtained by analyzing any finite region in the temperature field by the law of conservation of energy and Fourier’s law. For FVM, the calculation area is divided into several units, and the temperature interpolation function is set in each unit [28].

In the DOM, radiation intensity needs to be discretized in direction and space. For the calculation model in this paper, under the condition of the 2D rectangular coordinate system (x,y), RTE on the discrete direction ($\xi^{m}$,$\eta^{m}$) is shown in Equation (10). The boundary wall of opaque, diffuse emission and diffuse reflection gray body is used in this calculation model; thus, the corresponding boundary condition is shown in Equation (11). Finally, the discrete formula in each direction is discretized again in space by the finite difference method; thus, the radiation intensity is obtained.

(10) $$\xi^{m}\frac{\partial I^{m}}{\partial x}+\eta^{m}\frac{\partial I^{m}}{\partial y}=-\beta I^{m}+\kappa I_{b}(r)+\frac{\sigma_{s}}{4\pi }\left[\sum_{l=1}^{N_{\Omega }}w^{l}I^{l}\Phi^{m,l}\right]$$

(11) $$I_{w}^{m}=\varepsilon_{w}\frac{\sigma T_{w}^{4}}{\pi }+\frac{1-\varepsilon_{w}}{\pi }\sum_{n_{w}\cdot s_{l}<0}w^{l}I_{w}^{l}\left|n_{w}\cdot s_{l}\right|,\;n_{w}\cdot s_{m}>0$$

where l and m represent the l th and m th solid angle of space direction, respectively; $N_{\Omega }$ is the total number of solid angles with the space direction of 4π; $w^{l}$ is the integral weight coefficient; $\Phi^{m,l}$ is the scattering phase function after discretization; $I_{w}$ is the radiation intensity of the wall; $\varepsilon_{w}$ is emissivity of wall; $T_{w}$ is the temperature of the wall; $n_{w}$ is the normal vector of the wall.

In this paper, a CFD numerical simulation is performed to solve the govern equations. The commercial software, ICEM, is used to generate the mesh, and Equations (3)–(9) are solved by the commercial software, Fluent 19.2. Both the energy equation and RTE are discretized using the second order upwind scheme, whereas the unsteady item is discretized with the second order implicit scheme. The iterative process will stop when the residuals of the energy equation and RTE are less than 1.0 × 10^−8^.

## Figures and Tables

**Table 1:** The calculation conditions.

***T_C_* (K)**	**300 K**																		
*ε*	0.2					0.4				0.6			0.8				1.0		
*T_H_* (K)	600			900				1200			1500				1800			2100	
*β* (m^−1^)	5 × 10^1^	1 × 10^2^	2 × 10^2^		3 × 10^2^		5 × 10^2^		1 × 10^3^	2 × 10^3^		5 × 10^3^		1 × 10^4^		2 × 10^4^	5 × 10^4^		1 × 10^5^

**Table 2:** The physical properties.

	Parameter	ρ (kg/m^3^)	c (J/kg·K)	λ (W/m·K)	n	ε’
Material						
Metal		8470	812	28	1.6	28
Aerogel		300	1000	0.05	1.05	0.05

## Nomenclature

**Nomenclature**	
t	the optical thickness
$q_{R}$	the radiation emission from the coupling interface, ${W/m}^{2}$
$T_{p}$	the coupling interface temperature, T
$T_{C}$	the cold surface temperature, T
$T_{H}$	the boundary temperature, T
ΔT	the temperature difference across the MTPS, T
c	specific heat capacity, J/kg⋅K
n	the refractive index
$q_{t}$,$q_{c}$,$q_{r}$	total heat flux, conductive heat flux and radiative heat flux, ${W/m}^{2}$
$I_{b}(r)$	radiative intensity emitted by a black body, ${W/m}^{2}\cdot \mathrm{sr}$
I(r,s)	the radiative intensity of space position r and transmission direction **s,** ${W/m}^{2}\cdot \mathrm{sr}$
$q_{r,x}$, $q_{r,y}$	the components of radiative heat flux in x and y coordinates, ${W/m}^{2}$
l,m	the l th and m th solid angle of the space direction
$N_{\Omega }$	the total number of solid angles with a space direction of 4π
$w^{l}$	the integral weight coefficient
$I_{w}$	the radiation intensity of the wall, ${W/m}^{2}\cdot \mathrm{sr}$
$T_{w}$	the temperature of the wall, T
$n_{w}$	the normal vector of the wall
**Greek symbols**	
δ	thickness of the aerogel, m
δ’	thickness of the MTPS, m
β *,* κ *,* $\alpha_{s}$	extinction, absorption and scattering coefficients, 1/m
*λ*	thermal conductivity of conduction, W/m⋅K
ρ	density, ${\mathrm{kg}/m}^{3}$
σ	the Stefan–Boltzmann constant
ε’	inner emissivity of the aerogel
ε	emissivity of the coupling interface
$\varepsilon_{w}$	emissivity of the wall
Ω	solid angle, sr
$\Phi \left(s_{i},s\right)$	scattering phase function
ξ,η	direction cosine along the x coordinate and y coordinate, respectively
θ, φ	zenith angle and circumference angle, respectively
$\Phi^{m,l}$	the scattering phase function after discretization
